# Prevalence of Myopia among Children Attending Pediatrics Ophthalmology Clinic at Ohud Hospital, Medina, Saudi Arabia

**DOI:** 10.1155/2018/3708409

**Published:** 2018-11-07

**Authors:** Aisha Mohammed Alemam, Mohammed Hamad Aldebasi, Abdulkarem Rehmatullah, Rami Alsaidi, Ishraq Tashkandi

**Affiliations:** ^1^College of Medicine, Taiba University, Medina, Saudi Arabia; ^2^College of Medicine, Al Imam Mohammad Ibn Saud Islamic University (IMSIU), Riyadh, Saudi Arabia; ^3^MCPS, FICO(Lond), FRCSG, Paediatric Ophthalmologist, Ohud Hospital, Medina, Saudi Arabia; ^4^Msc, Ohud Hospital Medina, Saudi Arabia; ^5^MD, FRCSEdn, Consultant Pediatric Ophthalmologist, RTPD Ophthalmology Program, Ohud Hospital, Madina, Saudi Arabia

## Abstract

**Introduction:**

Around half of the visually impaired population has uncorrected refractive errors (URE), and myopia constitutes a high proportion of them. URE should be screened and treated early to prevent long-term complications. The aim of this study was to determine the prevalence of myopia among all patients attending a pediatric outpatient clinic at Ohud Hospital in Medina, Saudi Arabia (KSA).

**Method:**

This study was conducted using a convenience sample of all patients attending the clinic (1500 patients) aged between 3 and 14 years, and they were enrolled in the study during the period from May 2017 until September 2017.

**Result:**

Of 1215 subjects, only 43 (3.54%) were diagnosed with myopia. Out of the study participants, 56.8% were female and the mean age was 9.7 ± 3.6. Myopia was more prevalent in male participants than female participants (*n* = 525, 4%, *n* = 690, 3.1%, *p*=0.5). Low myopia was the most common form among the screened individuals. The level of myopia was associated with the degree of the strabismus angle. Approximately 22% of patients with myopia had >25° strabismus angle. There was a statistically significant association with both near work indoor and outdoor activities on weekends and the level of myopia.

**Conclusion:**

The prevalence of myopia among pediatrics patients in Medina is 3.54%. We hope that the results of this study will contribute to a better understanding of this public health issue in Saudi Arabia in order to implant a strict screening program for early detection and interventions to reduce the risk of further progression of visual impairment.

## 1. Introduction

Refractive errors (RE) are common health issue worldwide affecting a large proportion of the population, regardless of the sex, age, or race [1]. Fortunately, it can be easily measured, diagnosed, and managed either by spectacles or other refractive correction methods. If the RE is corrected inadequately or did not get treated at all, it may become a major cause of impaired vision or even blindness [2]. Uncorrected refractive errors (URE) represent almost half of the visually impaired population worldwide. Of those errors, myopia is the most commonly occurring [3]. Uncorrected vision should be screened early and treated immediately to minimize long-term complications on both children and adults. These long-term complications might include diminished quality of life and learning obstacles that might affect the educational level and the economic attainment.

Early diagnosis and treatment of RE are one of the easiest ways to reduce impaired vision or even blindness [4]. The World Health Organization (WHO) recently has issued a strategy to eliminate the avoidable visual disability and blindness which includes the correction of refractive errors [5].

The prevalence of myopia is attracting researchers around the world recently as many recent studies have reported dramatic increases over the last two decades. Worldwide, the prevalence of myopia reveals that more than 22% of the current total world population or 1.5 billion individuals are myopic [6]. Onset of the myopia commonly starts from the primary schoolchildren aged between 8 and 12 years [7]. It is typically progressing until the age of twenty due to the continuation of the eye growth.

The estimation of the prevalence of myopia is important for both health-care professionals and policy makers. This reflects the importance of the early screening and prompt management in order to avoid further visual impairment. Unfortunately, this public health issue has not been well defined in Saudi Arabia.

During the early childhood, the eye grows in a way that reserve the balance of the change in the corneal power, lens power, anterior chamber depth (ACD), and the axial length (AL) which keeps the refractive state towards emmetropia [8, 9]. Several earlier studies have shown that the ocular axial length (AL) increases parallel to the overall growth and development of the child [10, 11]. The AL and its interaction with the corneal radius of curvature play a key role in the emmetropization of the vision, and it has been found to be one of the major variables used to assess the refractive status of the eye [12, 13].

The purpose of this clinic-based study is to determine the prevalence of myopia among all patients attending the pediatrics outpatient clinic at Ohud hospital, Medina, Saudi Arabia, to contribute in providing a strong background to regulate the clinic for prevention of progression of the condition and raise awareness about early detection of myopia and guide the intervention in Medina.

## 2. Methodology

### 2.1. Study Design, Setting, and Sampling

This is a cross-sectional clinic-based study to estimate the prevalence of myopia in the pediatrics ophthalmology clinic in Ohud Hospital, Medina, Saudi Arabia, from May 2017 until September 2017. All patients who have attended the clinic (1500 patients) aged between 3 and 14 years were enrolled in the study. After initial examination, children (82 patients) presented with organic defects in the eye such as corneal opacity, lens opacity, and choroid and retinal disorders were excluded. A total number of 1215 of subjects between the ages of 3 and 14 years undergoes further examination.

### 2.2. Study Tool

**Figure 1:**
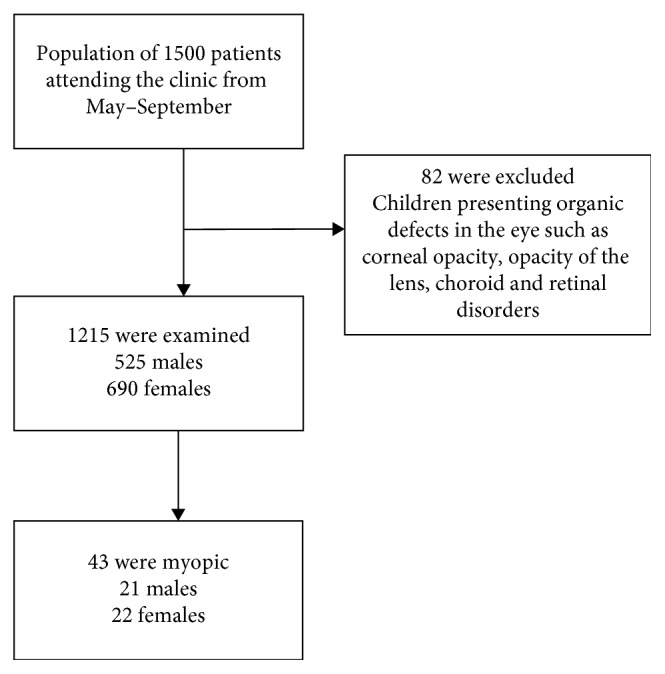
Flow diagram of the study population.

Patients' demographics and full medical history were documented. Every child underwent a complete initial ophthalmic examination by a qualified optometrist, including slit lamp examinations and ophthalmoscopes (Figure 1).

Subsequently, children were allocated into three groups according to their ages (less than 4 years, 5-6 years, and 7–14 years). For those who were 4 years old or younger, a cycloplegic refraction was performed by instilling 2 drops of 1% cyclopentolate with 10 minutes apart for each eye separately followed by a retinoscopy. For those who were 5-6 years old, a cycloplegic refraction was performed followed by a subjective refraction after three days. Cyclopentolate drops were used to dilate the pupil of the eye and relaxing the ciliary muscles and the refractive status was measured after 1 hour by a retinoscope and after 3 days when the mydriasis effect released, we perform the subjective refraction based on retinoscopy results. The Allen's chart picture was used to assess the visual acuity.

The older age group underwent visual acuity testing using a Snellen eye chart for each eye separately, according to the standard protocol, and underwent subjective refraction testing for each individual eye. The binocular balance test was used to determine the subjective refraction endpoint. The IOLMaster 700 was used to measure the axial length for both eyes for each myopic subject.

In this study, we have classified the patients according to the refractive errors by the spherical equivalent (SE): myopia was defined as an SE of −0.5 diopters (D) or less and hyperopia as an SE of 0.5 D or more. Further classifications included low, moderate, and high myopia as an SE of −0.5 to −3.0 D, −3.1 to −6.0 D, and less than −6.0 D, respectively. Ocular deviation was assessed using the cover-uncover test, with the correction, if any, both at 3 m and at 40 cm. The participants were asked to look straight ahead at a fixed target at near (40 cm) and distance (3.0 m) letter at 3 m. The test started with occluding the left eye for 3 seconds. The observer looked for any correcting movement of the uncovered eye. After testing the right eye, the left eye was tested in a similar manner. If there was no manifest misalignment of either eye, the cover was moved back and forth between both eyes, with 1-2 seconds between movements. Ocular alignment was assessed using the cover and uncover test. Cover testing was performed using fixation targets at near (0.5 m) and distance (4.0 m). The degree of tropia was measured using the Hirschberg corneal light reflex.

### 2.3. Statistical Analysis

Data were analyzed using Statistical Package for the Social Sciences (SPSS) software. The analysis compared the means and standard deviation for the continuous variables and proportions for the categorical variables. The chi-squared test was used to examine the differences among categorical variables, and the student *t*-test was used for continuous variables. The prevalence was calculated in percentage along with 95% confidence interval. A *P* value of 0.05 or lower was considered as a cutoff point for statistical significance.

### 2.4. Ethical Consideration

Official approval was obtained from the research regulatory authority at the hospital prior to the data collection. The parents or guardians provided a written informed consent assent prior to the study and were asked to fill the questionnaire about the eye health of their children.

## 3. Results

Among the 1215 included subjects, only 43 (3.54%) were having myopia. Of the study participants, 56.8% were female, and the mean age for them was 9.7 ± 3.6. The majority were Saudis and living in Madinah (95%) and (90%), respectively. Around 90% of the participants were eyeglass wearers, and 90% of them had been wearing them for more than one year (Table 1).

Table 2 shows the association between the eye position and the strabismus angle with the degree of myopia. More than half of the myopic patients have had normal eye position (62.8%). Low myopia was the most common form among the screened individuals. The level of myopia was associated with the degree of the strabismus angle (*P* < 0.002). Approximately, the overall proportion of patients with myopia having >25° strabismus angle was 25%.

Table 3 illustrates the amount of time spent doing indoor and outdoor activities and its association with the presence of myopia. There was a statistically significant association with near work-indoor and outdoor activities on the weekdays (*P* < 0.001^*∗*^) and (*P* < 0.0125^*∗*^), respectively. However, in the weekend, there was no association between either the near-indoor or outdoor activities and myopia.

Table 4 shows the relationship between the presence of myopia and other variables of interest. The visual acuity was associated with the level of myopia (*P* < 0.003). The level of myopia was associated with the axial length as well (*P* < 0.001). However, there was no relation between the level of myopia and the anterior chamber depth.

We found that 27.9% of the patients have paternal myopia (*P*=0.534) and 32.6% of the patients have maternal myopia (*P*=0.564).

Table 5 illustrates the amount of time that study participants spent doing outdoor activities on the weekdays and weekends and its association with the axial length and anterior chamber depth. There was a statistically significant association in both weekdays and weekend outdoor activities with the axial length and anterior chamber depth (*P*=0.010*∗*) and (*P* < 0.018*∗*), respectively, for weekdays and (*P*=0.046*∗*) and (*P* < 0.035*∗*), respectively, for weekends.

## 4. Discussion

This is a clinic-based cross-sectional study of patients attending a pediatric ophthalmology clinic at Ohud Hospital in Medina, Saudi Arabia, between 3 and 14 years of age.

In this study, the prevalence of myopia was 3.5% out of 1215 respondents. It was more prevalent in males than in females (4% and 3.1%, respectively), which differs from other studies. A study conducted in 1995 of schoolchildren in Taiwan reported a lower prevalence and lesser degree of myopia among boys [14]. Other researchers in Finland reported a lower prevalence in boys compared to girls [15], and the possible explanation might be that girls at the primary school level tend to read and write more than boys. The subsequent increase in near-indoor work predisposes them to develop myopia. Further studies are needed to clarify such propositions.

Taking into consideration the difference in the definition of myopia, the prevalence of myopia found in this study is slightly similar to other studies conducted in different regions of Saudi Arabia. The prevalence was previously reported to be 5.8% in Qassim [16] and 2.5% in Riyadh [17].

In comparison with other countries, the prevalence of myopia in our study population is considered to be comparable to the prevalence in Australia 2% [18], Iran 4.3% [19], Ethiopia 2.6% [20], Macedonia 1.6% [21], and Nigeria 2.7% [22]. However, it was significantly lower than the prevalence in north India 79.5% [23], US 41.9% [24], and South Korea 47% [25]. The difference was slightly lower than the prevalence in Morocco 6.1% [26] and China 8% [27], as shown in Table 6.

The differences can be partially attributed to the differences in the study setting. It might also be attributed to the genetic susceptibility to myopia that varies across different races and cultural settings.

Previous studies [28–31] showed an increase in the prevalence of myopia parallel to the increase of age. In our study, the prevalence of myopia was not significantly higher in the range between 11 and 14 years than other younger age groups (*n* = 31.3, 4.7%, *P*=0.3).

In regard to physical activities, indoor activities, such as watching TV, reading, playing video games, and doing homework, have been proposed to be in charge of the remarkable increment in the prevalence of myopia [32]. A study conducted in Australia among school children demonstrated that myopic children performed significantly more near work [33]. Our study demonstrated a statistically significant association with outdoor activities and axial length and anterior chamber depth in both weekdays and weekends (*P*=0.010*∗*) and (*P* < 0.018*∗*), respectively, for the weekdays and (*P*=0.046*∗*) and (*P* < 0.035*∗*), respectively, for the weekends, which contradict the result of other studies [18, 34, 35].

However, the association between outdoor activities and prevention of the onset and the progression of myopia is still not fully clear [36]. Several studies have recently suggested that greater time spent in outdoor activities might be associated with the reduction in myopia prevalence [18, 37]. While the exact mechanism of this association is not well recognized, some theories have been proposed, as “light-dopamine theory,” which stated that the exposure to the sunlight during outdoor activities stimulate the release of dopamine neurotransmitter from the retina which has been suggested to have the ability to inhibit elongation of the axial length of the eye [18, 34, 35]. Some studies showed that exposure to high light intensities can retard myopia in animals as chicks [38, 39] and monkeys [40]. Studies showed that the exposure to a light level of 15,000 lux for 5 hours per day produced significantly lower myopia and shorter axial length, whilst exposure to 500 lux did not retard eye growth and myopia in chicks [39]. The degree of protection was directly proportional to the increasing light levels [38]. The protective effect was more evident when exposed to a light intensity of about 10,000 lux, and this was significantly associated with higher vitreous dopamine concentration and lesser myopia development in chicks [41]. Moreover, the sunlight in the outdoor area would lead to pupil constriction resulting in the increased depth of focus and decrease image blur [41, 42].

A previous study showed that myopia appeared to be more frequently seen in children with myopic parents [43]. In this study, the parental myopia was assessed to test the hypothesis of inherited susceptibility to develop myopia, and no significant evidence was found to prove this hypothesis. We found the axial length to be associated with the degree of myopia (*P* < 0.0001); however, there was no relation between the level of myopia and the anterior chamber depth. Previous studies found that eyes with higher myopia tend to have a deeper anterior chamber [44, 45]. Other studies found that individuals with hyperopia tend to have a shorter AL and myopia tend to have a longer AL [46]. Zadnik et al. found that hyperopic eyes (22.62 + 0.76 mm) have significantly (*P* < .001) shorter axial length than myopic eyes 25.16 + 1.23 mm [47]. Numerous studies [48, 49] showed a significant association between myopia and exotropia; however, our result showed no major association between them. Most of the patients were having normal eye position 62.8% (*P*=0.179). Although low myopia was the most common form among the screened individuals, the level of myopia was associated with the degree of the strabismus angle (*P* < 0.002). The exact mechanism of the association between myopia and exotropia is not fully understood. Further studies are needed to prove this association and to clarify the link between them.

### 4.1. Limitation and Recommendation

Our study had some limitations. First, it was a clinic-based study; therefore, not all children in Medina city were included in the sample. We cannot generalize the results on the population of Medina. Second, the study was performed only within five months' duration, a longer duration would provide better knowledge on the prevalence of myopia, and we would have been able to follow the patient in order to study the impact of the growth of the eyeball and the progression of myopia.

Professional-based screening programs are recommended to address the issue of uncorrected refractive error in children in order to provide an early detection and to begin prompt treatment.

## 5. Conclusion

The prevalence of myopia among pediatrics patients in Medina is 3.5%. We believe that estimating the prevalence of myopia is important because it opens a new ground for policy making, program planning, and the establishment of health promotion interventions regarding ocular-related complications. We hope that the results of this study will contribute to a better understanding of this public health issue in KSA and help to highlight the need for screening, early detection, and subsequent interventions to reduce the risk of further progression of visual impairment.

## Figures and Tables

**Table 1:** Demographic characteristics of the children included in the screening.

Variables		*N* (%)	No. of myopic patients	Prevalence of myopia	Chi-square	
Sex	Male	525 (43.2)	21	4.00	*X* ^2^	*P* value
	Female	690 (56.8)	22	3.19	0.331	0.565

Age	3–6	327 (26.9)	9	2.75		
	7–10	507 (41.7)	16	3.16	2.205	0.332
	11–14	381 (31.3)	18	4.72		
	Total	1215 (100%)	43			

**Table 2:** The association between severity of myopia with eye position and strabismus angle.

Variables			Myopia			Total	Chi-square	
			Low	Moderate	High		*X* ^2^	*P* value
Eye position	Normal	*N*	21	13	20	54	8.912	0.179
		%	63.6	54.2	69.0	62.8		
	Exophoria	*N*	9	8	3	20		
		%	27.3	33.3	10.3	23.3		
	Exotropia	*N*	3	3	4	10		
		%	9.1%	12.5%	13.8%	11.6%		
	Esotropia	*N*	0	0	2	2		
		%	0.0	0.0	6.9	2.3		

Strabismus angle	5°	*N*	8	2	0	10	24.999	0.002^*∗*^
		%	66.7	18.2	0.0	31.3		
	10°	*N*	1	3	0	4		
		%	8.3	27.3	0.0	12.5		
	15°	*N*	0	1	5	6		
		%	0.0	9.1	55.6	18.8		
	20°	*N*	1	1	2	4		
		%	8.3	9.1	22.2	12.5		
	>25°	*N*	2	4	2	8		
		%	16.7	36.4	22.2	25.0		

^*∗*^Significant at *P* value less than 0.05.

**Table 3:** Near work-indoor and outdoor activities and its association with the presence of myopia.

Variables	On school weekdays		On weekends	
	*N*	%	*N*	%
Near work-indoor activities (reading, watching TV, playing video games, and/or writing homework)				
<1 hour	11	25.6	6	13.9
1-2 hours	14	32.6	8	18.6
≥3 hours	18	41.9	29	67.4
*P* value	0.42		<0.001^*∗*^	

Outdoor activities (football, running, and/or swimming)				
Not at all	14	32.5	12	27.9
<1 hour	11	25.6	7	16.3
1-2 hours	10	23.3	5	11.6
≥3 hours	8	18.6	19	44.2
*P* value	0.62		0.0125^*∗*^	

**Table 4:** The relation between myopia and other variables of interest.

Variables	Myopia			*P* value
	Low	Moderate	High	
	Mean ± SD	Mean ± SD	Mean ± SD	
Age	9.5 ± 3.5	9.5 ± 3.3	10.1 ± 4.0	0.7
Visual acuity in logMAR	.25 ± 0.25	.49 ± 0.3	0.46 ± 0.3	0.003^*∗*^
Axial length in mm	24.3 ± 2.2	24.3 ± 1.5	26.2 ± 1.7	0.000^*∗*^
Anterior chamber depth in mm	3.48 ± 0.3	3.5 ± 0.4	3.5 ± 0.3	0.9

^*∗*^Significant at *P* value less than 0.05.

**Table 5:** Outdoor activities and its association with the axial length and anterior chamber depth in weekdays and weekends.

	Not at all		<1 hour		1-2 hours		≥3 hours		*P* value
	Mean	SD	Mean	SD	Mean	SD	Mean	SD	
*Outdoor activities (football, running, and swimming) on school weekdays*									
Axial length	24.3	1.2	24.6	2.2	25.4	2.6	26.3	1.6	0.01^*∗*^
Anterior chamber depth	3.4	0.3	3.4	0.4	3.6	0.2	3.6	0.2	0.01^*∗*^

*Outdoor activities (football, running, and swimming) on weekends*									
Axial length	24.4	1.3	24.5	2.5	26.3	3.1	25.2	1.8	0.046^*∗*^
Anterior chamber depth	3.4	0.3	3.4	0.5	3.5	0.2	3.6	0.2	0.035^*∗*^

**Table 6:** Prevalence of myopia in different regions worldwide.

Country	Sample size	Studied age group (years)	Prevalence of myopia (%)
Saudi Arabia-Qassim (Aldebasi Yousef H)	5176	6–13	5.8%
Saudi Arabia-Riyadh (Al-Rowaily Mohammad A)	1319	4–8	2.5%
Nigeria-Aba (Atowa UC et al.)	1197	8–15	2.7%
Macedonia-Tetovo (Mahmudi E. et al.)	119	3–9	1.6%
Ethiopia-Addis Ababa (Jafer K et al.)	570	7–15	2.6%
Morocco (Anera et al.)	545	6–16	6.1%
Iran-Shiraz (Yekta et al.)	2130	5–15	4.3%
India-North India (Saxena Rohit et al.)	9884	5–15	79.5%
China-Guangzhou (He M et al.)	**5053**	**5–15**	**8%**
Australia-Sydney (Rose KA et al.)	**1735**	**6–12**	**2%**
US-California (Theophanous et al.)	60,789	**5–19**	**41.9%**
South Korea (Jang JU et al.)	1079	**8–13**	**47%**

## Data Availability

The data used to support the findings of this study are available from the corresponding author upon request.
